# Preparation and Evaluation of Novel Emodin-loaded Stearic Acid-g-chitosan Oligosaccharide Nanomicelles

**DOI:** 10.1186/s11671-020-03304-1

**Published:** 2020-04-25

**Authors:** Xiaohong Jiang, Mingxing Ma, Mingjuan Li, Shihong Shao, Hong Yuan, Fuqiang Hu, Jianwen Liu, Xuan Huang

**Affiliations:** 1 College of Medical, Jiaxing University, Jiaxing, China; 2 Qingdao Fifth People Hospital, Qingdao, China; 3 College of Pharmacy, Zhejiang University, Hangzhou, China; 4 East China University of Science and Technology, Shanghai, China

**Keywords:** Emodin, CSO-SA, CSO-SA/EMO preparation, Antitumor

## Abstract

The purpose of this study was to prepare and characterize emodin-loaded stearic acid-g-chitosan oligosaccharide (CSO-SA/EMO) and to evaluate its antitumor activity in vitro. In this study, stearic acid-g-chitosan oligosaccharide was used as a carrier and its physicochemical properties were determined by different methods. Cell uptake behavior was examined using FITC-labeled stearic acid-g-chitosan oligosaccharide. CSO-SA/EMO was prepared using ultrasonication and dialysis. Particle size, surface potential, entrapment efficiency, and drug release behavior were studied in vitro. The effects of CSO-SA/EMO on gastric cancer cells were investigated using MTT assay and flow cytometry. Results showed CSO-SA/EMO particle size was larger and potential was smaller than that of stearic acid-g-chitosan oligosaccharide. The 12 h micellar uptake by MGC803 and BGC823 cells was sufficient, and the micelles were able to abundantly accumulate at lesion sites in mice thus achieving good passive EPR targeting. MTT and cell cycle arrest assays showed CSO-SA/EMO-enhanced antitumor activity significantly towards MGC803 and BGC823 cells compared with that of free emodine. Tumor volume, hematoxylin and eosin staining, and terminal deoxynucleotide transferase dUTP nick-end labeling assay proved CSO-SA/EMO had a significant antitumor effect on tumor tissues in vivo. In conclusion, the ultrasonication-dialysis method provided a simple and effective method for preparing CSO-SA/EMO. The delivery of emodine using a micelle system improved its antitumor effects effectively.

## Introduction

Emodin (EMO) is a natural anthraquinone derivative that is extracted mainly from traditional Chinese herbs such as rhubarb, cuspidatum, and multiflorum. Traditional Chinese medicine (TCM) is widely used in clinical research because of its low toxicity, few side effects and low cost [1].

Studies have shown EMO has broad pharmacological activity, including immune-suppression, anti-pertussis, anti-hypertensive, anti-inflammatory, antibacterial, and anticancer activities. EMO has been found to inhibit the growth of cancer cells [2–4] and to regulate related genes to control tumor cell apoptosis, tumorigenesis, cell proliferation, invasion, and metastasis [5–9]. Studies have shown EMO can inhibit diverse cancer cells, such as human colorectal adenocarcinoma cells, hepatocarcinoma cells, lymphoid leukemia cells [10], and human tongue cancer SCC-4 [11]. EMO can inhibit the proliferation of tumor cells in gastric, breast, and prostate cancers [7, 12]. However, EMO exhibits no cytotoxic effects on normal cells such as normal human gingival fibroblasts [13], human bronchial epithelial cells [14], and human mammary cells [15]. These show EMO exhibits selective cytotoxicity toward tumor cells compared with normal cells.

Chitosan, a natural polysaccharide, is the deacetylated form of chitin. This natural polymer has excellent water solubility, bio-functionality, blood compatibility, and microbial degradability characteristics, and is known for its various biomedical applications. We degraded high molecular weight chitosan (450 kDa) using chitosanase under acidic conditions to obtain low molecular weight chitosan oligosaccharide (CSO, 18 kDa). CSO can strongly penetrate cell membranes [16] and stearic acid (SA) can enter nucleus through the glycoprotein pathway. CSO was hydrophobically modified with SA using carbodiimide (EDC) as a coupling reagent to synthesize amphipathic stearic acid-g-chitosan oligosaccharide (CSO-SA).

Although EMO has extensive biological activity, its anthraquinone structure is poorly soluble in water. Because therapeutic drugs are transported through the bloodstream, their solubility affects their absorption and distribution directly. CSO-SA grafts can self-assemble in an aqueous solution to form nanomicelles that are hydrophobic inside and hydrophilic outside. We dispersed the nanomicelles from the gathered state using an ultrasound probe. Because both EMO and CSO were hydrophobic, EMO was encapsulated at the center of the micelles.

Several model drugs are applied to CSO-SA, which requires molecular weight, structure, and hydrophobicity of model drugs. Existing studies include curcumin [17], doxorubicin [18], lamivudine stearate [19], and oxaliplatin [20] which can improve antitumor effects significantly. We explored optimal loading conditions of CSO-SA/EMO. CSO-SA/EMO creates new dosage forms with higher solubility and utilization efficiency. CSO-SA/EMO can provide ideas for selecting model drugs or carriers, and clinical application of micelles.

## Experimental Materials and Methods

### Experimental Materials

BALB/C+/nu male nude mice were obtained from the Zhejiang University Experimental Animal Center. Low-differentiation gastric cancer cell lines MGC803 and BGC823 were purchased from the ATCC cell bank. RPMI-1640 culture medium and FBS were obtained from Hangzhou Holly Leaf Biotechnology Company. EMO, MTT, FITC, Hoechst 33342, DiR, trinitrobenzene sulfonic acid, and pyrene were supplied by Sigma Aldrich. Zhejiang University furnished CSO-SA. Other reagents purchased were of AR grade.

### Determination of the CMC and ^1^H NMR Spectra of CSO-SA

In this report, critical micelle concentration (CMC) of CSO-SA was determined by fluorescence spectrophotometry using pyrene as a fluorescent probe. Various concentrations of CSO-SA solution were added to a pyrene acetone solution after which the acetone was evaporated overnight. The emission spectra and peak values of pyrene in CSO-SA solutions of different concentrations were analyzed by fluorescence spectrophotometry. The first peak (*I*_1_ = 374 nm) and the third peak (*I*_3_ = 385 nm) of the spectrum were recorded. We plotted the logarithmic concentration (Log C) as the abscissa and *I*_1_/*I*_3_ as the ordinate and calculated the CMC for the polymer micelles.

CSO and CSO-SA were dissolved in D_2_O at a concentration of 10 mg/ml. ^1^H NMR spectra were recorded, compared, and analyzed for characteristic CSO and CSO-SA peaks.

### Detected Amine Substitution Degree

The amine substitution degree (SD%) was detected using the trinitrobenzene sulfuric acid (TNBS) method.

Different concentrations of CSO and CSO-SA solutions were prepared after which 4% NaHCO_3_ and 0.1% TNBS were added successively. After incubation at 37 °C for 2 h in a water bath, 2 mol/L hydrochloric acid was added. Absorbance was measured at 344 nm by ultraviolet-visible spectrophotometry after 30 min of ultrasonication. A standard curve was drawn and the SD% of CSO-SA sample was calculated.

### CSO-SA Particle Size and Potential

A 1.0 mg/ml CSO-SA solution was prepared and micelles were dispersed completely with an ultrasonic cell disruptor probe. The particle size and potential of CSO-SA were determined with particle size and surface potential analyzer.

### Cell Uptake of CSO-SA

FITC and CSO-SA solutions were mixed, stirred overnight, and transferred to a dialysis bag. Un-reacted FITC and absolute ethanol were removed by dialysis with deionized water for 24 h. Finally, FITC labeled CSO-SA (FITC-CSO-SA) solution with a concentration of 1.0 mg/mL was obtained.

MGC803 and BGC823 cells were used as target cells to examine cell uptake of CSO-SA. Based on cell proliferation rate, cells were seeded in 24-well plates and cultured overnight until they became completely adherent. Eighty μL of FITC-CSO-SA solution was then added at a set time point. Cells were incubated with 10 μL of Hoechst 33342 (1 mg/mL) for 15 min to stain cell nucleus. Uptake of CSO-SA by FITC-CSO-SA was detected by laser scanning confocal microscopy.

### In Vivo Distribution of CSO-SA

The distribution of CSO-SA in vivo was determined by DiR fluorescent dye staining. CSO-SA/DiR solution was prepared by dialysis.

Six-week-old male nude mice were used as the experimental model. Nude mice were inoculated subcutaneously with 1 × 10^8^/mL MGC803 cells. CSO-SA/DiR was administered via the tail vein at a tumor cell volume of approximately 200 mm^3^. The mice were then anesthetized at set time points. Timed distribution of CSO-SA/DiR in vivo was recorded using a small animal live body imager (wavelength range, 580-700 nm, exposure time 1000 ms).

### Detection of EMO Concentration by HPLC

EMO concentration was determined by HPLC. EMO was applied to an Eclipse XDB-C18 packed column (4.6 × 250 mm, 5 μm) with a protection column (4.6 × 10 mm, 5 μm). Column temperature was set at 30 °C, flow rate was 1.0 mL/min, detection wavelength was 254 nm, and the injection volume was 20 μL. The mobile phase was methanol/0.1% phosphoric acid (85:15, v/v). EMO concentration was plotted as the abscissa and the peak area as the ordinate. Standard curves of EMO were drawn to determine the optimum linear range. All HPLC-injected samples were filtered through a 0.45-μm organic filter to protect the column.

### Preparation of CSO-SA/EMO

In this study, EMO was encapsulated with an ultrasonic probe. An initial formulation ratio of 10% (EMO: CSO-SA, w/w) was used as a starting point. The EMO solution was slowly added in a drop-wise manner to the micellar solution in an ice bath. The ultrasonic probe was then applied for 20 cycles (400 W, work 2 s, stop 3 s).

The drug-loaded micelles were transferred to a dialysis bag (MWCO, 3.5 kDa) and dialyzed against deionized water for 24 h to remove ethanol from the solvent. Pure CSO-SA/EMO was obtained by centrifugation of the dialysate to remove free EMO.

### Properties of CSO-SA/EMO (Particle Diameter Potential, TEM, EE%)

The particle size and surface potential of CSO-SA/EMO were measured with particle size and surface potential analyzer.

A 0.1 mg/ml CSO-SA/EMO solution was added dropwise onto a carbon film covered copper wire, stained with 2% phosphotungstic acid and dried. The morphology and particle size of CSO-SA/EMO were observed by transmission electron microscopy.

Entrapment efficiency (EE%) and drug loading (DL%) of CSO-SA/EMO were detected by organic solvent extraction and HPLC. To a 200 μL sample of CSO-SA/EMO micellar solution, 1.8 mL of methanol was added to disperse the micelles and extract the drug. The EMO concentration was measured as C_EMO_. Another 400 μL of CSO-SA/EMO micellar solution was placed in ultrafiltration centrifuge tube and centrifuged (12,000 rpm, 5 min) to obtain the supernatant. The EE% and DL% were calculated according to the following formulas:

$$ \mathrm{EE}\%=\left(10\times {C}_{\mathrm{EMO}}-C\right)\times V/{M}_{\mathrm{EMO}}\times 100\% $$

$$ \mathrm{DL}\%=\left(10\times {C}_{\mathrm{EMO}}-C\right)\times \mathrm{V}/\left[\left(10\times {C}_{\mathrm{EMO}}-C\right)\times V+{M}_{\mathrm{CSO}-\mathrm{SA}}\right]\times 100\% $$

where *C*_EMO_ is the concentration of EMO in micelles, *C* is the EMO concentration of the drug-loaded micelles after ultrafiltration centrifugation, *V* is the volume of drug-loaded micelle subjected to dialysis, *M*_EMO_ is the amount of EMO administered during drug loading, *M*_CSO-SA_ is the mass of CSO-SA.

### Evaluation of Drug Release In Vitro

Drug release from CSO-SA/EMO was investigated using PBS (pH 7.2) as a release medium. One milliliter of drug-loaded micelle solution was placed in a 3.5 kDa dialysis bag sealed at both ends then placed in an appropriate release medium containing tube. The dialysis bag was placed in a 37 °C horizontal thermostat shaker. Samples were taken at set time points and replaced with the same volume of fresh release medium. The content of EMO in the samples was determined by HPLC and the cumulative amount of EMO released was calculated.

### Cytotoxicity of CSO-SA/EMO

The survival rates of gastric cancer cells treated with CSO-SA/EMO, CSO-SA, and EMO were detected by MTT assay. Cells were seeded in 96-well plates at a concentration of approximately 10^5^/ml. CSO-SA/EMO, CSO-SA, and EMO were added at different concentrations. After incubation for different time periods, 20 μL of MTT working solution was added. After 4 h of incubation, 200 μL of DMSO was added and the optical density (OD) of the solution at 570 nm was measured by microplate reader.

### Effect of CSO-SA/EMO on the Cell Cycle

Cells from the two cell lines were seeded in a 6-well plate at a density of 10^5^/mL. After 12 h of culture, CSO-SA, CSO-SA/EMO, and EMO were added and incubated for 24 h. The cells were then digested, collected, and washed. A 500 μL of PI staining solution was then added to each cell sample, the cell pellet was slowly resuspended, and the cells were incubated in the dark at 37 °C for 30 min. Red fluorescence was detected by flow cytometer at an excitation wavelength of 488 nm. DNA content was analyzed by the FlowJo software.

### Antitumor Effects of CSO-SA/EMO Evaluated by In Vivo and Histological Analyses

Animal experiments were performed according to the Guidelines for Animal Care and Use Committee, Zhejiang University. MGC803 cells (1 × 10^6^) were injected subcutaneously into the right anterior flank of male nude mice at 5 to 6 weeks of age. Tumors were allowed to grow to a diameter of approximately 5 mm. At that point, the mice were randomly divided into a control group, an EMO injection group and a CSO-SA/EMO injection group, with 3 animals in each group. All mice were injected intravenously with the relevant reagents via the tail vein once a day for 2 weeks. An electronic Vernier caliper was used to measure the long (a) and short (b) diameters of the tumor (b). The tumor volume was calculated according to the formula *V* = a × b^2^/2. The body weight of each mouse was recorded after which the tumors were collected for histologic analysis. Tumor inhibitory rate was determined in the tumor tissues following hematoxylin and eosin (HE) staining. Terminal deoxynucleotide transferase dUTP nick-end labeling (TUNEL) was performed to detect cellular apoptosis in terminal ileum tissue using an in situ cell death detection kit according to the manufacturer’s instructions.

## Results and Discussion

### CMC and ^1^H NMR Spectra of CSO-SA

**Fig. 1:**
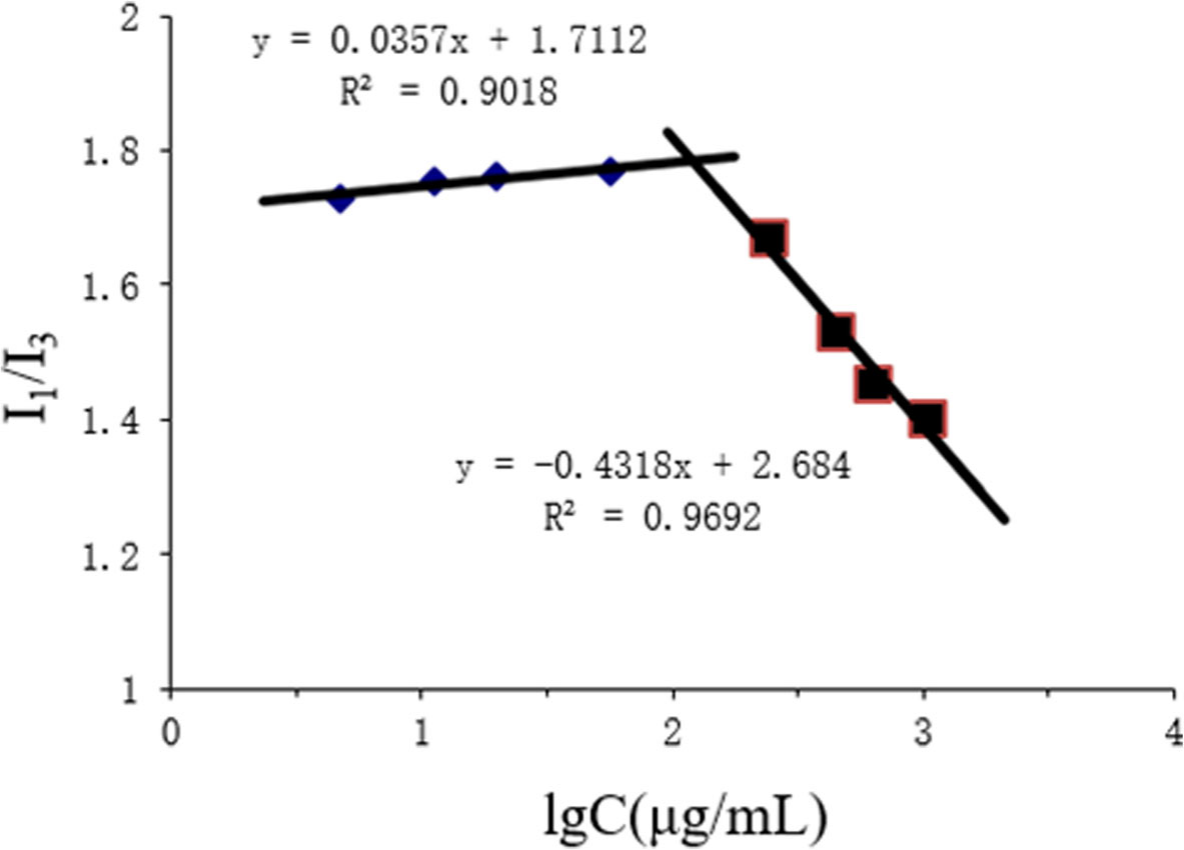
Variation of fluorescence intensity ratio (*I*_1_/*I*_3_) versus logarithm concentration of CSO-SA

The amphiphilic polymers self-assembled into micelles in hydrophilic medium. Lower CMC resulted in greater micelle formation. This was beneficial for the maintenance of micelles structure after intravenous administration. When CSO-SA formed micelles, the pyrene fluorescent probe could easily enter the hydrophobic core of the micelles increasing fluorescence intensity of the charged pyrene which increased the intensity of the emission spectrum. At this point, *I*_3_ increased significantly faster than *I*_1_, thus, the *I*_1_/*I*_3_ fluorescence intensity ratio decreased abruptly. A significant breakpoint could be observed in the *I*_1_/*I*_3_ plot and Log C values (Fig. 1). Inflection point calculations showed that CMC was 179.02 μg/mL. The smaller the CMC, the stronger the ability to form micelles and the stronger the resistance to dilution which improved micelle structure protection following intravenous administration.

EDC was used as a crosslink coupling agent to react with the carboxyl group of SA and form the active intermediate -OO-acyl isourea derivative. This can react with the primary amine group of CSO to form an amide bond. The structure of CSO-SA was determined and confirmed based on the nuclear magnetic resonance proton spectrum. ^1^H NMR (400 MHZ, D_2_O) δ 1.06 (m, CH2), 1.02 (m, CH3) correspond to the methylene proton, and methyl proton of SA respectively.

### Substitute Degrees of Amino Group

The SD% of CSO-SA is the percentage of stearic acid-substituted amino groups on chitosan. TNBS reacted with free amino groups on chitosan and formed trinitrobenzene derivatives with a UV absorption at 344 nm. The standard curve of the trinitrobenzene derivative of chitosan was determined by UV absorption at 344 nm. The SD% of CSO-SA was calculated to be 9.3 ± 8%. This confirmed that CSO and SA had been successfully linked based on their graft ratio.

### CSO-SA, CSO-SA/EMO Particle Size and Potential

Table 1 shows that the Z-average of CSO-SA was 139.3 ± 2.2 nm. PDI was 0.179 ± 0.03 indicating a relatively uniform dispersion. Compared with CSO-SA, the Z-average and PDI of CSO-SA/EMO increased. After loading EMO, the surface positive charge of CSO-SA/EMO was higher than that of CSO-SA.

**Table 1:** Physicochemical properties of CSO-SA and CSO-SA/EMO

Formulation	Z-Average (nm)	PDI	Zeta (mV)
CSO-SA	139.3 ± 2.2	0.179 ± 0.03	34.9 ± 1.0
CSO-SA/EMO	238.3 ± 3.1	0.327 ± 0.05	25.8 ± 1.4

### Cell Uptake

**Fig. 2:**
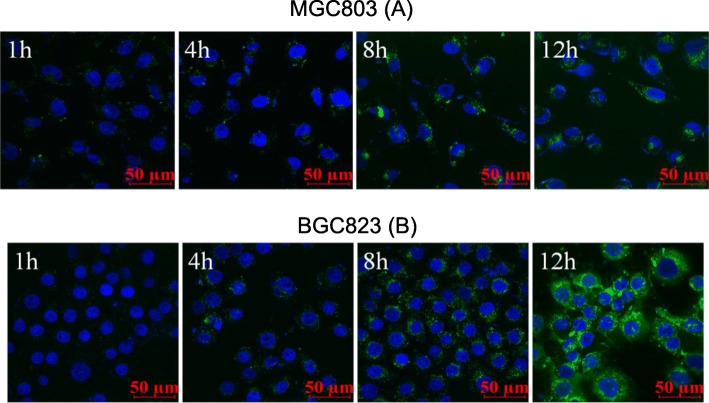
In vitro cellular time-dependent uptake of CSO-SA micelles in MGC803 (**a**) and BGC823 (**b**) cells for 1, 4, 8, 12 h, respectively (blue, Hoechst 33342; green, FITC; scale bar = 50 μm)

FITC did not affect the physicochemical properties of CSO-SA. We obtained FITC-CSO-SA by grafting fluorescein FITC into CSO-SA. After cells were stained with Hoechst 33342, the uptake of FITC-CSO-SA cells was observed using confocal laser scanning microscopy. We found that the uptake of MGC803 gradually increased with increasing time and FITC fluorescence gradually increased. The micellar uptake of BGC823 was similar to MGC803 in a time-dependent manner. FITC-CSO-SA had good cell penetrating ability and was distributed evenly in cytoplasm of gastric cancer cells (Fig. 2).

### Distribution of CSO-SA In Vivo

**Fig. 3:**
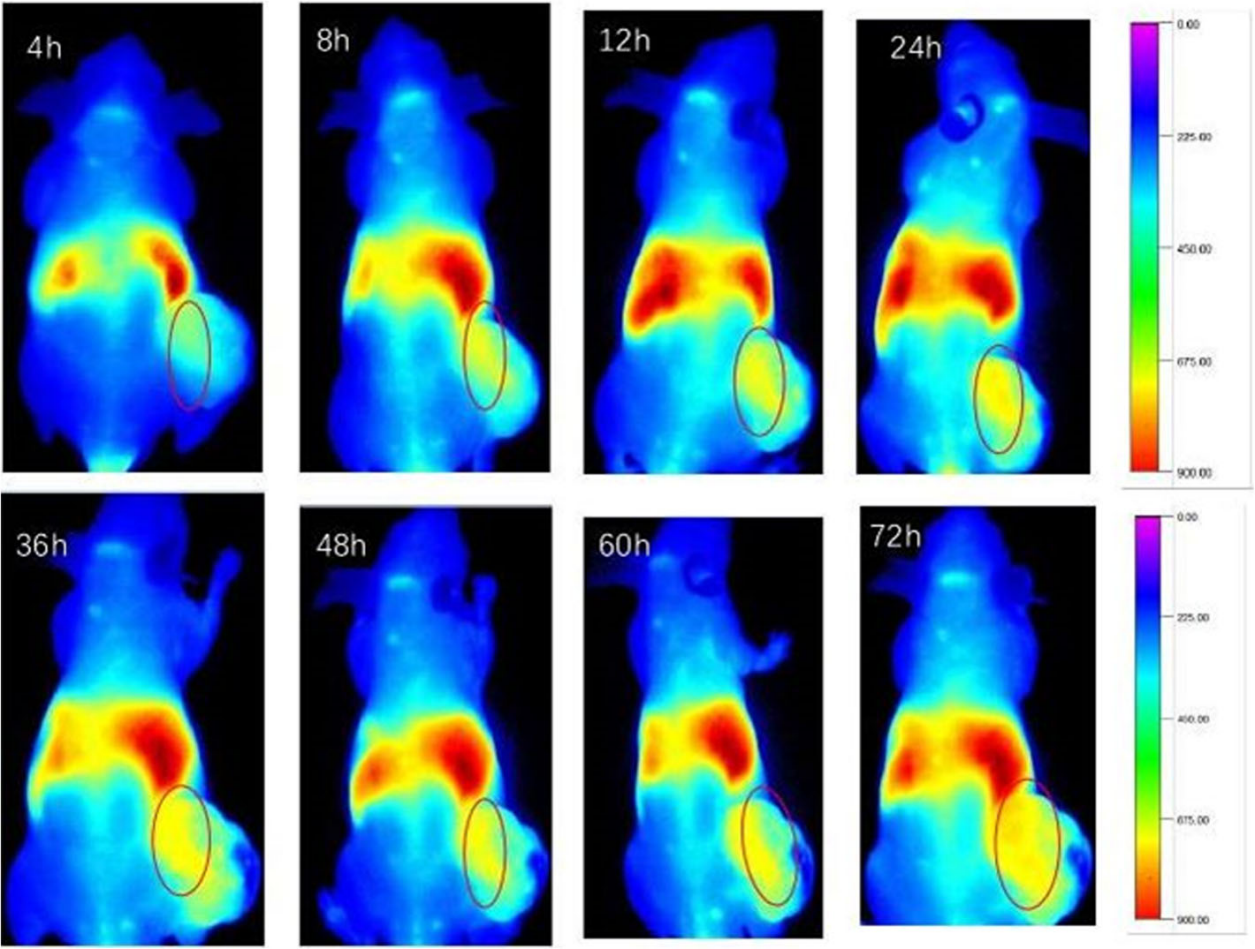
Whole body image of CSO-SA/DiR at different times after i.v. injection

Near-infrared (NIR) imaging technology is advantageous for penetrating biomaterials and tissues. After CSO-SA/DiR was injected into the tail vein of nude mice, distribution of CSO-SA/DiR was observed and recorded at different times using a small animal living body imager. As shown in Fig. 3, the distribution of CSO-SA/DiR was similar to other micelles at 4 h following tail vein injection and was mainly distributed in the liver, spleen, and tumor tissues. The distribution of nanomicelles in tumor increased gradually in intensity with increasing time. This indicated CSO-SA graft had a good passive targeting ability mainly due to the EPR effect of nanomicelles (Fig. 3).

### Preparation of CSO-SA/EMO

#### The Influence of Different pH Environments on Drug Loading

**Fig. 4:**
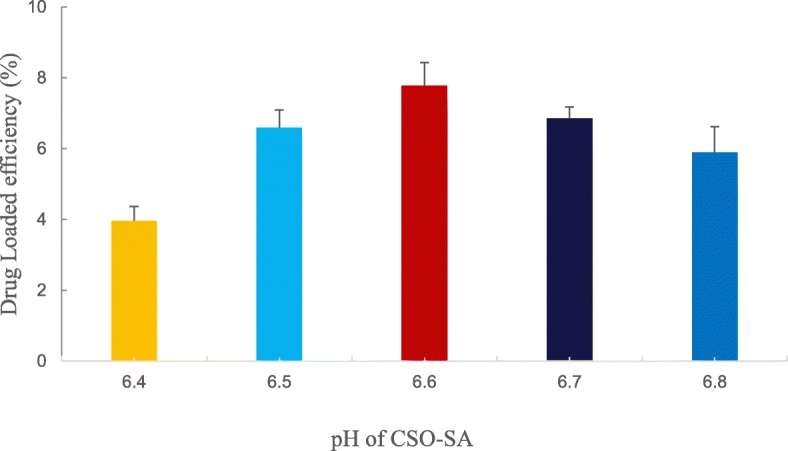
Effects of different pH values of CSO-SA grafts on drug loading. Data are presented as means ± SD (*n* = 3)

We prepared a CSO-SA solution and adjusted the pH of the CSO-SA to investigate the effect of pH on drug loading. As shown in Fig. 4, CSO-SA had good drug-loading capacity and exhibited a parabolic trend in the pH 6.4-6.8 range. The highest level of drug loading was seen at pH 6.6 making this value the optimal pH for drug loading (Fig. 4).

#### Encapsulation Efficiency, TEM, and Drug Loading

**Fig. 5:**
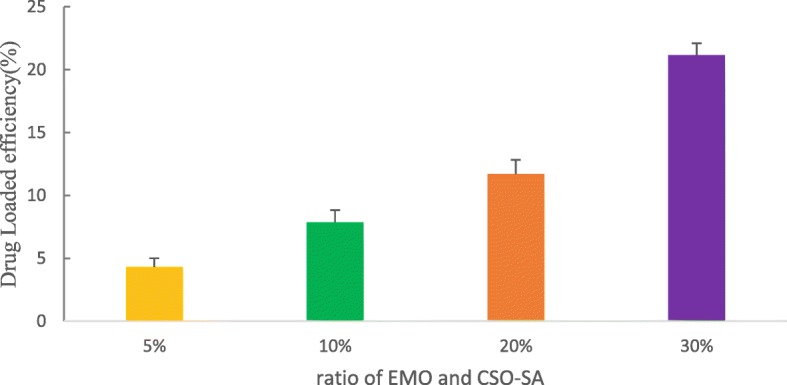
The influence of different ratios of EMO and CSO-SA (5-30%) on drug loadings. Data are presented as the means ± SD (*n* = 3)

CSO-SA self-assembles into shell-core nanocomposite micelles. The hydrophobic moieties spontaneously form hydrophobic core structures to repel the hydrophilic media. This structure provides a key vehicle for drug loading because EMO can easily be encapsulated in the hydrophobic core due to its hydrophobic structure. We investigated the effect of CSO-SA on the loading capacity of EMO by varying the dosage of EMO. As shown in Fig. 5, with increasing EMO dosage, the amount of drug loading increases. The loading rate of CSO-SA reached as high as 21.16% when the ratio was 30% (Fig. 5).

CSO-SA/EMO was magnified 30,000 times with a transmission electron microscope. Nanomicelles shape and size were observed and recorded.

### EMO Release from CSO-SA/EMO

The drug-loaded micelle solution was added to a 3.5 kDa dialysis bag with PBS (pH 7.2) being used as a release medium. Each time a sample was removed it was replaced with the same volume of fresh release medium.

**Fig. 6:**
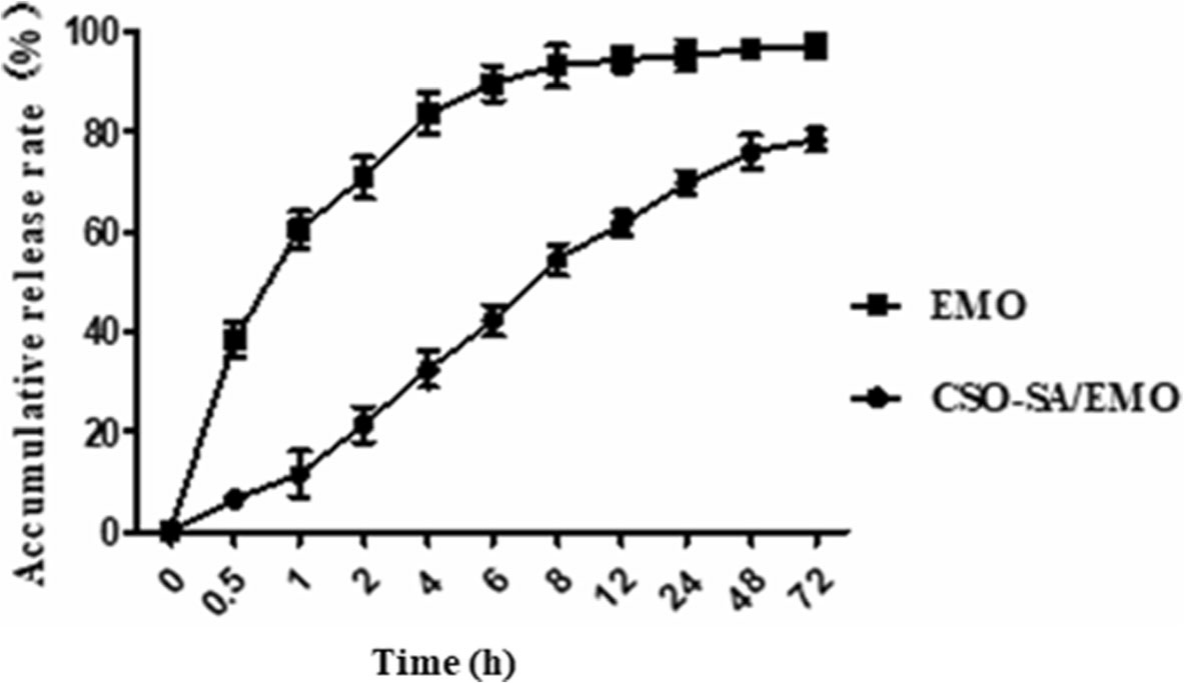
EMO release profile from CSO-SA/EMO over 72 h. Error bars in the graph represent the standard deviations (*n* = 3)

The concentration of EMO at different time points was determined by HPLC, after which the cumulative release percentage was calculated. As shown in Fig. 6, CSO-SA/EMO exhibited a significant sustained-release effect compared with free EMO. These results show that the release percentage of free EMO was 38.4% at 0.5 h while the release percentage of EMO from CSO-SA/EMO was approximately 6.6%. At 4 h, the release percentage of free EMO was 83.7% and the release percentage of CSO-SA/EMO was approximately 32.5%. Within 72 h, the release of free EMO reached 97.2% while the release of EMO from CSO-SA/EMO was 78.4%. EMO was released from CSO-SA/EMO drug-loaded micelles in two main ways: through EMO dissociation from the micellar nucleus and by penetration from the micelle core into the release medium.

### Toxicity of CSO-SA/EMO to Gastric Cancer Cells

**Fig. 7:**
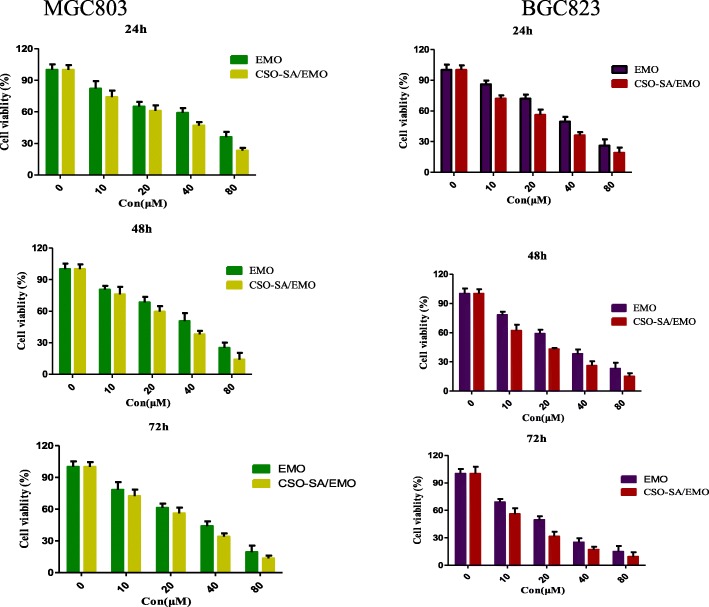
Cell viability of MGC803 and BGC823 gastric cancer cells treated with CSO-SA, EMO, and CSO-SA/EMO for 24 h, 48 h, and 72 h. Data are presented as the means ± SD (*n* = 3)

The MTT assay is a standard method for detecting cell viability. The cytotoxicity of free EMO, CSO-SA, and CSO-SA/EMO to MGC803 and BGC823 gastric cancer cells was measured using the MTT assay. It showed that EMO and CSO-SA/EMO could inhibit MGC803 and BGC823 cells in a dose- and time-dependent manner. However, at the same concentration, CSO-SA/EMO had a higher inhibitory effect on MGC803 and BGC823 cells compared to the free EMO solution. The IC_50_ values (Table 2) of EMO and CSO-SA/EMO for gastric cancer cells were calculated. The IC_50_ of CSO-SA/EMO was significantly lower than that of EMO at each time point (24 h, 48 h, 72 h). The IC_50_ values of CSO-SA showed that CSO-SA was a safe biological carrier with little biological toxicity which confirmed that the cytotoxicity of CSO-SA/EMO was caused by EMO (Fig. 7).

**Table 2:** The IC_50_ values (μmol/L) of EMO and CSO-SA/EMO against gastric cancer cells

Cell	Formation	24 h	48 h	72 h
MGC803	EMO	50.41	39.19	30.39
	CSO-SA/EMO	37.72	26.32	21.72
BGC823	EMO	39.96	27.15	20.13
	CSO-SA/EMO	22.37	18.35	11.91

Table 2 and Fig. 7 showed the cytotoxicity of CSO-SA/EMO drug delivery system was significantly higher than that of free EMO. However, when the release of CSO-SA/EMO and free EMO in an in vivo environment was simulated, the release of free EMO reached 38.4% at 0.5 h while the release of CSO-SA/EMO was only 6.6%. Overall, the concentration of free EMO was higher than that of CSO-SA/EMO at each time point.

This phenomenon is easily explained. Although CSO-SA/EMO released EMO relatively slowly, the antitumor effect of CSO-SA/EMO does not slow or decline. Many studies have shown nanodrug delivery systems can improve the antitumor activity of chemotherapy drugs [21].

First, although free EMO exhibits an initial burst of activity, EMO cannot be efficiently taken up by the cells whereas CSO-SA greatly accelerated cell uptake through endocytosis or phagocytosis.

Second, when CSO-SA/EMO closes to cell membranes, the interaction between nanoparticles and cell membranes can affect the structure and properties of nanomicelles as well as the function of biological macromolecules such as ion channels. Therefore, the attachment of CSO-SA/EMO to the cell membranes does not result in a simple physical adsorption but can change the dynamic balance of cell and thereby further promote the cytotoxicity of CSO-SA/EMO.

Finally, free EMO has a hydrophobic anthraquinone structure resulting in poor distribution in blood. The CSO-SA/EMO drug delivery system increases solubility of EMO and improves dissolution leading to a higher molecular concentration in surrounding cells.

### Detection of Cell Cycle Arrest by FCM

Cell cycle analysis is an important aspect of the clinical treatment of malignant tumors. Application of cycle-specific drugs to tumor cells during drug-sensitive periods has been shown to have great efficacy. Although ethidium bromide analogs cannot penetrate normal cells, they can stain cell nuclei by penetrating damaged cell membranes. PI-embedded double-stranded DNA produces red fluorescence and the fluorescence intensity is proportional to the level of double-stranded DNA. DNA content was determined by flow cytometry (FCM) after DNA staining with PI. Cell cycle distribution and apoptosis were analyzed based on DNA content distribution.

Studies have shown that EMO affects cell cycle distribution [11, 22]. FCM analysis showed that CSO-SA/EMO and EMO could block MGC803 and BGC823 cells in the G2/M phase of the cell cycle.

**Fig. 8:**
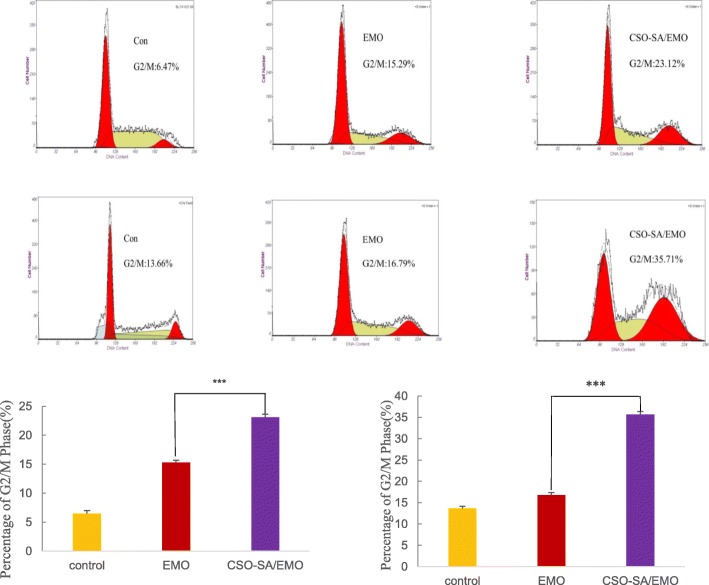
Cell cycle distributions of MGC803 and BGC823 cells treated with EMO and CSO-SA/EMO for 24 h. Student’s *t* test was calculated and the data are presented as the means ± SD (*n* = 3), (*p < 0.05, ^**^p < 0.005, ^***^p < 0.001)

MGC803 cells were exposed to the same concentration (20 μM) of CSO-SA, free EMO and CSO-SA/EMO solution for 24 h. The percentage of MGC803 cells in G2/M phase was found to be 8.95%, 15.29%, and 23.12% respectively. Control group percentage was 6.47%. The percentages of BGC823 cells in G2/M treated with 20 μM CSO-SA, free EMO, or CSO-SA/EMO under the same conditions were 14.25%, 16.79%, and 35.71% respectively which were higher values than in the control group (13.66%). These data showed that CSO-SA/EMO blocked the G2/M phase of MGC803 and BGC823 cells more efficiently and significantly than free EMO at the same concentration. The CSO-SA showed no difference from the controls (Fig. 8).

### Antitumor Effect of CSO-SA/EMO In Vivo

**Fig. 9:**
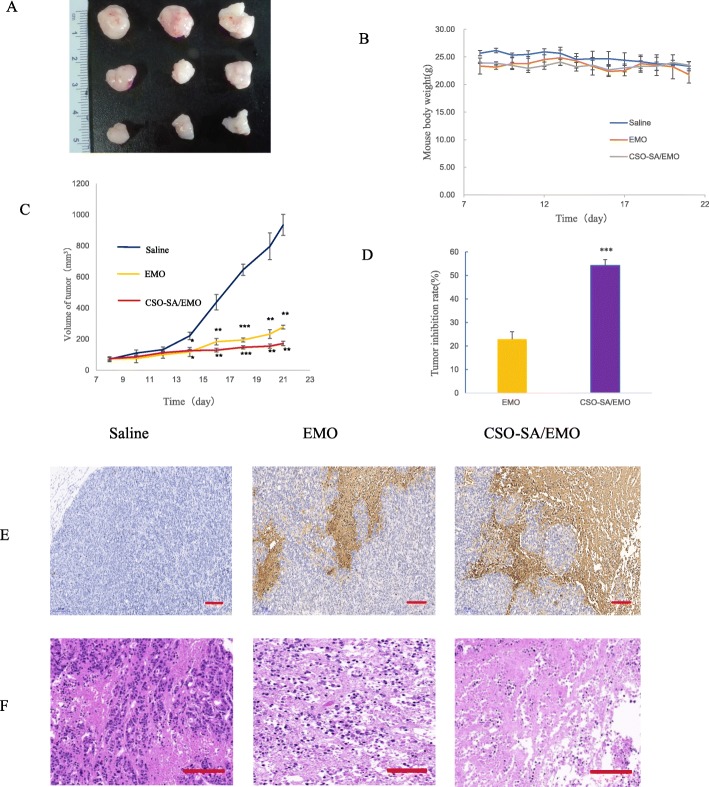
Antitumor effect of CSO-SA/EMO in vivo. **a** Tumor shape in each group. **b** Mouse body weight in each group. **c** Tumor volume in each group. **d** Tumor inhibition rate. **e** Representative images of TUNEL-stained tumor tissue from each group (scale bar = 200 μm). **f** Representative images of HE-stained tumor tissue from each group (scale bar = 200 μm)

To further study the antitumor effect of CSO-SA/EMO, the tumor inhibition rate and cellular apoptosis levels were evaluated in vivo. The results showed that both CSO-SA/EMO and EMO had a notable inhibitory effect on tumor growth (Fig. 9a). The tumor inhibition rate of the CSO-SA/EMO group was three times that of the EMO group (Fig. 9d). The weight of mice in each group did not change significantly (Fig. 9b). This showed that both CSO-SA/EMO and EMO were relatively safe. Morphological alterations and apoptosis in tumor tissues of each group showed that CSO-SA/EMO had significant antitumor effects (Fig. 9e, f).

## Conclusions

In this study, we detected structure, CMC, SD%, particle size, and potential of CSO-SA with different methods. Results prove amphiphilic CSO-SA performed well as a carrier. Fluorescence intensity of gastric cancer cells showed CSO-SA had been taken up rapidly within 12 h. Nude mice were used as a model to study the distribution of CSO-SA within 72 h. Over time, CSO-SA distribution in lesion became more concentrated exhibiting a good passive targeting effect.

CSO-SA/EMO was prepared with ultrasound-dialysis method. CSO-SA had strong drug-loading capacity. When environment pH was set at 6.6, the level of drug loading reached 21.6% at a 30% formulation ratio. Compared with CSO-SA, CSO-SA/EMO had bigger particle size and zeta potential. The shape of CSO-SA/EMO could be recorded directly using TEM. The release of EMO from CSO-SA/EMO was 78.4% within 72 h, it indicated a smooth and continuous process in body. Considering the antitumor effect of CSO-SA/EMO compared with free EMO, we confirmed it with various experiments.

CSO-SA toxicity to gastric cancer cells was detected by MTT assay. The results showed that CSO-SA was a safe biological carrier. Compared with that of free EMO, CSO-SA/EMO significantly enhanced the antitumor activity against gastric cancer cells. MGC803 and BGC823 cell cycle could be arrested in the G2/M phase more effectively by CSO-SA/EMO. Tumor volume, HE staining, and TUNEL assay proved more significant antitumor effect of CSO-SA/EMO than free EMO.

Based on above results, CSO-SA is both biocompatible and safe carrier. CSO-SA/EMO in this study utilizes a new and effective dosage formulation for the treatment of cancer and exhibits good passive targeting effect in vivo.

## Supplementary information

**Additional file 1: Figure S1.**^1^H NMR spectra of CSO and CSO-SA.

**Additional file 2: Figure S2.** Transmission electron microscope observation of CSO-SA/EMO (magnification = 30000, scale bar = 1 μm).

## Data Availability

All data generated or analyzed during this study are included in this published article.

## Abbreviations

CSO-SA: Chitosan oligosaccharide-stearic acid
EMO: Emodin
FITC: Fluorescein isothiocyanate
MTT: Thiazolyl blue tetrazolium bromide
TEM: Transmission electron microscope
^1^H NMR: Proton nuclear magnetic resonance
EPR: Enhanced permeability and retention
TCM: Traditional Chinese medicine
CMC: Critical micelle concentration
Log C: Logarithm of concentration
