# Supplementary material for: Preparation and Evaluation of Novel Emodin-loaded Stearic Acid-g-chitosan Oligosaccharide Nanomicelles
Source: Nanoscale Res Lett. 2020 Apr 25;15:93. doi: 10.1186/s11671-020-03304-1 (PMC7183521; doi:10.1186/s11671-020-03304-1)
Supplement: Supplementary file 1 — Additional file 1: Figure S1.1H NMR spectra of CSO and CSO-SA. [file 11671_2020_3304_MOESM1_ESM.ppt]

## Slide 1
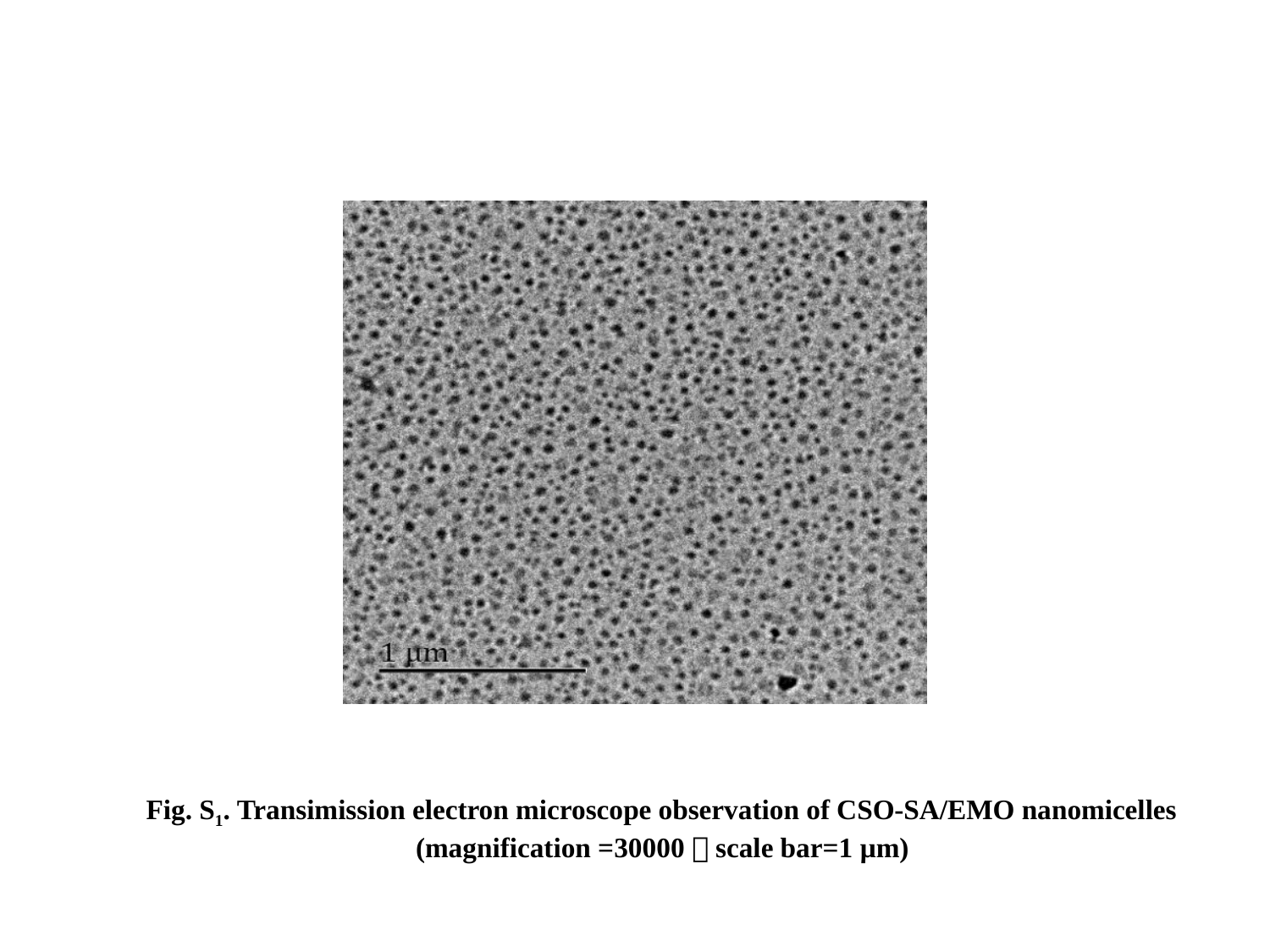

Fig. S1. Transimission electron microscope observation of CSO-SA/EMO nanomicelles
(magnification =30000，scale bar=1 μm)
