# Supplementary figures and images for: Preparation and Evaluation of Novel Emodin-loaded Stearic Acid-g-chitosan Oligosaccharide Nanomicelles
Source: Nanoscale Res Lett. 2020 Apr 25;15:93. doi: 10.1186/s11671-020-03304-1 (PMC7183521; doi:10.1186/s11671-020-03304-1)

## Slide 1
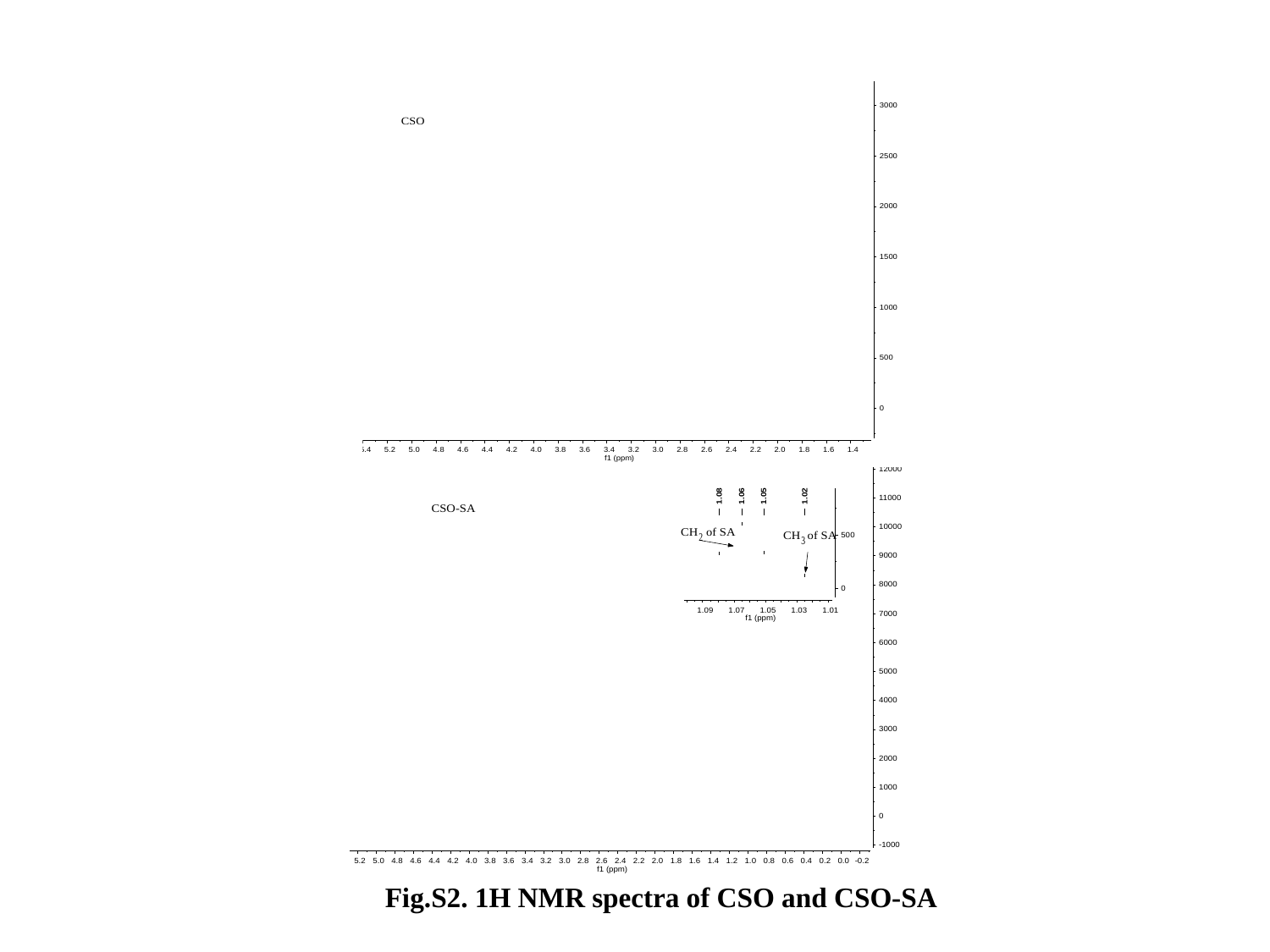

Fig.S2. 1H NMR spectra of CSO and CSO-SA

Supplement: Supplementary file 2 — Additional file 2: Figure S2. Transmission electron microscope observation of CSO-SA/EMO (magnification = 30000, scale bar = 1 μm). [file 11671_2020_3304_MOESM2_ESM.ppt]
